# Chromosome karyotype and stability of new synthetic hexaploid wheat

**DOI:** 10.1007/s11032-021-01253-w

**Published:** 2021-09-24

**Authors:** Yajuan Wang, Siwen Wang, Xiujuan Jia, Zengrong Tian, Yongfu Wang, Changyou Wang, Hong Zhang, Xinlun Liu, Jixin Zhao, Pingchuan Deng, Wanquan Ji

**Affiliations:** 1 College of Agronomy, Northwest A & F University, Yangling, 712100 Shaanxi China; 2 State Key Laboratory of Crop Stress Biology for Arid Areas, Yangling, 712100 Shaanxi China; 3 Shaanxi Research Station of Crop Gene Resources & Germplasm Enhancement, Ministry of Agriculture, Shaanxi, 712100 China

**Keywords:** Synthetic hexaploid wheat, Chromosome karyotype, FISH, Mc-GISH, Resistance to stripe rust

## Abstract

Synthetic hexaploid wheat offers breeders ready access to potentially novel genetic variation in wild ancestral species. In this study, we crossed MY3478 (2*n* = 4*x* = 28, AABB) as the maternal parent with the stripe rust–resistant SY41 (2*n* = 2*x* = 14, DD) as the paternal parent to construct the new hexaploid wheat line NA0928 through natural allopolyploidization. Agronomic traits and the cytology of the S_8_–S_9_ generations of NA0928 were analyzed. Abundant variation in agronomic traits was observed among each strain of NA0928 in the S_8_ generation. Agronomic traits were superior in strains resistant to stripe rust compared with those of highly susceptible strains. The rank order of the coefficients of variation were tiller number (55.3%) > spike length (15.3%) > number of spikelets (13.9%) > plant height (8.7). Number of tillers and spike length are important traits in wheat breeding to improve yield. Cytological observation and fluorescence in situ hybridization showed that the chromosome number and configuration showed rich variation among NA0928 strains in the S_9_ generation. Chromosome number ranged from 36 to 44. Variation in chromosome karyotype was detected in the A and B subgenomes. Meiotic chromosome behavior in pollen mother cells and multicolor genomic in situ hybridization revealed that two new synthetic hexaploid wheat strains showed genetic stability; one strain was resistant to stripe rust and developed multiple tillers, and the other strain was susceptible to stripe rust, but both showed improved thousand-kernel weight (TKW) weight and produced multiple tillers. The two strains will be valuable germplasm resources for use in wheat breeding.

## Introduction

Owing to the extensive use of elite parents for directional selection in wheat breeding programs, the gene pool of wheat cultivars has become increasingly narrow and an increasing percentage of genetic diversity has been lost (Mohammadi et al. 2021). Many studies have shown that the narrow genetic pool not only limits improvement of crop yield and quality, but also increases the vulnerability of crops to biotic and abiotic environmental stresses. The declining genetic diversity has become a bottleneck that limits the ability to achieve major advances in wheat breeding (Yang et al. 2021).

Synthetic hexaploid wheat is considered to be a valuable germplasm resource for the introduction of unique genes for agronomically important traits into bread wheat from closely related or progenitor species in the primary gene pool (Ogbonnaya et al. 2013). Primary synthetic hexaploids are of poor agronomic value, difficult to thresh, generally tall, low yielding, and frequently of poor quality, but they harbor considerable genetic diversity for resistance to diverse biotic and abiotic stresses (Trethowan and Mujeeb-Kazi 2008). Novel synthetic hexaploid wheat has been widely used to improve wheat yield and quality (Li et al. 2018), and tolerance to abiotic stresses, including drought (Rattey and Shorter 2010), boron (Emebiri and Ogbonnaya 2015), and waterlogging (Villareal et al. 2001). Synthetic wheat showed 8–30% improvement in potential yield compared with the best local cultivars grown under the same conditions with irrigation or rainfed conditions (Dreccer et al. 2007). Ogbonnaya et al. (2007) observed that more than 80% of synthetic derivatives of wheat showed superior performance in terms of 1000-seed weight, yield, and other agronomic traits compared with the recurrent parent. Li et al. (2011) reported that one high-yield potential locus, *Barc1183*, derived from synthetic hexaploid wheat was detected in “Chuanmai 42.” This locus had positive effects on increasing tiller number per plant, number of effective spikes, grain number per square meter, harvest index, and grain production rate, and the average yield was increased by 8.92% compared with that of “Chuannong 16” in six environments, therefore, *Barc1183* from SHW is a candidate locus in high-yield breeding of wheat. Several stripe rust resistance genes harbored in synthetic hexaploid wheat have been reported*.* The gene *Yr48* was derived from the wheat genotype PI 610,750, a synthetic spring wheat with the pedigree Croc1/*Ae. tauschii* (Synthetic 205)//Kauz. With regard to stripe rust disease severity, the main effect of *Yr48* accounted for 10% of the observed variation in the field and was associated with an average 63% reduction in disease severity (Chen and Kang, 2017). The gene *YrC142* confers all-stage resistance to stripe rust and originated from the synthetic wheat CI142; it provides resistance to six Chinese races of the causal pathogen *Puccinia striiformis* f. sp. *tritici* (*Pst*) (Wang et al. 2009). Therefore, new synthetic hexaploid wheat has been the focus of increasingly detailed research.

Although euploidy is the cause of natural hexaploidy in wheat, newly synthesized allohexaploid wheat are associated with widespread and persistent whole-chromosome aneuploidy, and no significant differences in fitness between euploid and aneuploid wheat are observed, at least under laboratory conditions. Zhang et al. (2013) showed that structural mutations are rarely detected in new synthetic hexaploid wheat. However, variation in chromosome number frequently accompanies allopolyploidization. Occasionally, a “hidden” aneuploid carrying the same chromosome number as euploids but exhibiting dosage compensation is identified. Bian et al. (2020) reported that stress-tolerant progeny with increased euploidy frequency were able to be selected in early generations from synthetic wheat that survived the stimulation of allohexaploidization. Therefore, a certain frequency of cytologically stable euploids can be obtained.

In the present study, we identified the karyotypes of inbred progenies of S_8_–S_9_ individual plants, in the genetic background of synthetic hexaploid wheat NA0928, with known chromosome constitutions and agronomic characters using cytological methods and fluorescence in situ hybridization (FISH). Our ultimate aim is to select disease-resistant euploid strains and enhance the diversity of genetic resources available for wheat genetic breeding.

## Materials and methods

### Plant materials

*Triticum turgidum* L. subsp. *dicoccum* (Schrank ex Schübl.) Thell. accession MY3478 (2*n* = 4*x* = 28, AABB), and *Aegilops tauschii* Coss. accession SY41 (2*n* = 2*x* = 14, DD) were used as parents. The synthetic hexaploid wheat population NA0928 was obtained by selfing the MY3478/SY41 triploid S_0_ hybrid plants. Neither embryo rescue nor hormone treatment was applied to the S_1_ seeds. The S_1_ plants were self-pollinated to produce S_2_ to S_9_ generations. By repeatedly selfing, S_8_–S_9_ plants carrying different chromosome numbers were preserved to form separate lines. All plants were grown in the field at the College of Agronomy, Northwest A&F University, Yangling City, Shaanxi Province, China. Hui Xianhong (HXH) was the susceptible control for evaluation of stripe rust resistance at adult stages. *Triticum urartu* Thum. accession XM943 (2*n* = 2*x* = 14, AA) and *Ae. speltoides* Tausch accession SY183 (2*n* = 2*x* = 14, SS) were used for multicolor genomic in situ hybridization (mc-GISH) as probes and for blocking. The accessions XM943, SY183, MY3478, and SY41 were provided by Dr Li-Hui Li and Xin-Ming Yang from the Institute of Crop Science, Chinese Academy of Agricultural Sciences.

### Evaluation of agronomic traits and stripe rust resistance

The morphology of S_8_–S_9_ lines of NA0928, and the parents MY3478 and SY41, were assessed at the physiological maturity stage in 2016 and 2017 in the field. Each strain was planted in one row, with row length 1 m, and between-row spacing 0.1 m. Agronomic traits such as plant height, spike length, and number of grains per spike, were recorded before harvest.

The stripe rust resistance test was conducted in the field at adult stages. On an afternoon during wheat jointing, the physiological races CYR32, CYR33, and CYR34 were mixed with talc in proportions 1:400, the mixture of strains was used to inoculate plants by shaking the powder onto the leaves and 100% relative humidity for 12–14 h with plastic film. Infection types (Its) were recorded 14–15 days after inoculation. When the susceptible control HXH was fully infected, MY3478, SY41, and each plant of the S_8_ generation in NA0928 were assessed. The reaction to the mixed *Pst* races was scored as infection type on a scale from 0 to 4 (Ma et al. 1995) as follows: 0 and 0; were assessed to be immune and almost immune, respectively, 1 was regarded to be resistant, 2 was considered to be moderately resistant, and 3 and 4 were recorded as susceptible.

### Cytological observations

The S_8_ generation seeds were harvested and five seeds from each individual were selected to determine the chromosome number. Cytogenetic observation of mitosis in root tip cells was performed as previously described by Wang et al. (2016a, 2016b), including indoor germination, root collection, and observation of the chromosome number.

Field-grown plants of the S_9_ generation raised from individuals in the preceding generation containing 42 chromosomes were selected at the appropriate stage of development. Young inflorescences were immediately fixed in ethanol:chloroform:acetic acid (6:3:1, v/v/v). The subsequent procedure followed the method described previously (Wang et al. 2016a, 2016b).

Root tip cells and pollen mother cells with complete chromosome numbers in the S_8_–S_9_ generation of NA0928 were observed and photographed using an BX-43 microscope (Olympus, Tokyo, Japan) equipped with a DP80 camera.

### FISH and multicolor-GISH

Fluorescence in situ hybridization was conducted using synthetic oligonucleotide probes as described by Tang et al. (2014) and Zhao et al. (2016). The probes Oligo-pSc119.2 (green) and Oligo-pTa535 (red) were synthesized and labeled with FAM or TAMRA by Shanghai Invitrogen Biotechnology Co. Ltd (Shanghai, China) and used for FISH as described by Tang et al. (2014).

For mc-GISH analysis, XM943 DNA was labeled with Texas Red-5-dUTP (Invitrogen) and SY41 DNA was labeled with Texas Green-5-dCTP (Invitrogen) (2014). SY183 DNA was used as the blocker. Chromosomes were counterstained with 4, 6-diamidino-2-phenylindole (DAPI). Fluorescent signals were scanned and photographed with an Olympus BX53 microscope equipped with a Photometrics SenSys CCD DP80 camera (Olympus, Tokyo, Japan). (Wang et al. 2016a, 2016b).

## Results

### Agronomic traits and stripe rust resistance of new synthetic wheat NA0928 in the S_8_ generation

The S_8_ generation of NA0928 comprised 24 strains, and 141 plants were examined in the field in 2016. The main agronomic characters of the parents and S_8_ lines of NA0928 showed that the average of MY3478 were 145 cm tall, the number of spikelets per spike was 25, and all plants were highly susceptible to stripe rust infection. Plants of the paternal parent SY41 were generally short, with abundant effective tillers (up to 50), and strong resistance to stripe rust. The ranges of plant height from 14 strains (No. 1–14) of NA0928 were 94.5–119.0 cm, spike length 10.3–14.1 cm, number of spikelets per spike 15.0–19.0, effective tiller number 5.5–17.5, and the plants were highly susceptible to stripe rust. The ranges of plant height from ten strains (No. 14–24) of NA0928 were 115.0–137.0 cm, spike length 11.5–17.0 cm, number of spikelets per spike 21.0–23.0, effective tiller number 20.6–30.7, and plants were highly resistant to stripe rust. Thus, the agronomic traits of stripe rust-resistant strains were superior to those of highly susceptible strains (Table 1). A comprehensive summary of the agronomic traits in the field is presented in Table 2. Abundant variation in agronomic traits was observed between each strain. The rank order of the coefficients of variation was effective tiller number (55.3%) > spike length (15.3%) > number of spikelets per spike (13.9%) > plant height (8.7%). More effective tiller number and longer spike length traits are valuable for utilization in wheat breeding.

**Table 1:** The main agronomic characters in S8 lines of the synthetic wheat

Material	No. strain	Number plants	Plant height (cm)	Spike Lengths (cm)	Spikelets/spike	Tillers	Stripe rust resistance
MY3478		10	145.0	11.3	25.0	11.2	4
SY41		5	80.0	4.5	3.0	50.0	0
NA0928-1–2-2–3-5–2-1–1	1	4	112.5	13.4	17.0	12.3	4
− 3	2	4	114.8	14.1	18.8	9.0	4
− 4	3	6	114.3	13.3	17.3	12.0	4
− 5	4	5	117.2	13.0	18.2	12.0	4
− 6	5	8	121.7	13.5	18.7	12.2	4
NA0928-2–1-9–1-2–2-2–1	6	7	94.5	10.3	15.0	5.5	4
− 2	7	5	118.0	14.0	18.5	14.5	4
− 3	8	5	101.5	11.3	15.5	17.5	4
− 4	9	5	115.3	13.3	17.7	10.0	4
− 5	10	7	110.0	12.4	18.0	12.0	4
NA0928-2–1-10–2-1–3-1–1	11	7	119.3	13.2	17.4	12.7	4
− 3	12	3	113.0	13.0	19.0	12.0	4
− 4	13	8	106.6	11.5	16.8	12.7	4
− 5	14	4	107.3	12.0	17.5	8.8	3
NA0928-2–1-10–4-1–1-1–1	15	7	115.0	16.0	22.0	30.7	0
− 2	16	7	123.0	16.0	23.0	27.4	0
− 3	17	6	131.0	17.0	23.0	22.5	0
− 4	18	6	127.7	16.4	22.0	24.0	0
NA0928-2–1-10–4-1–3-9–1	19	7	137.0	16.9	22.5	21.0	0
− 2	20	7	127.0	15.2	21.0	22.7	0
− 5	21	4	128.0	16.0	21.0	22.0	0
− 6	22	4	132.0	15.2	21.3	20.6	0
− 7	23	7	131.7	16.3	22.7	24.4	0
− 9	24	8	128.3	15.0	20.3	21.0	0
Total	24	141					

All data is average in each strain per plant

**Table 2:** The average value of agronomic traits and variation coefficient in S8 lines of the synthetic wheat

Traits	Variation range	Average	Standard deviation	Coefficient of variation (%)
Plant height (cm)	88.0–139.0	118.4	10.3	8.7
Spike lengths (cm)	8.2–18.0	13.7	2.1	15.3
Spikelets/spike	12.0–24.0	18.8	2.6	13.9
Tillers	5.0–55.0	18.7	10.4	55.3

### Chromosome number of the S_9_ lines of NA0928

Chromosome counts in root tip cells from 600 seeds revealed variation in the somatic cell configurations of different synthetic S_9_ lines of NA0928, all seeds in S_9_ are bulk harvested from S_8_. The chromosome number ranged from 36 to 44, among which euploids (2*n* = 42) accounted for 51.7% and aneuploids for 48.3%. Discounting the plants containing 42 chromosomes, the aneuploids with chromosome numbers ranging from 36 to 44 accounted for 0.3% (2*n* = 36), 1.0% (2*n* = 37), 2.0% (2*n* = 38), 2.7% (2*n* = 39), 6.6% (2*n* = 40), 26.0% (2*n* = 41), 8.7% (2*n* = 43), and 1.0% (2*n* = 44), respectively, of the total number of individuals.

The chromosome number varied within the NA0928 strains. The chromosome number was determined in root tip cells from 126 seeds from the S_9_ strain NA0928-1–2-2–3-5–2-1: two seeds had 36 chromosomes, five seeds had 37 chromosomes, nine seeds had 38 chromosomes, six seeds had 39 chromosomes, 10 seeds had 40 chromosomes, 18 seeds had 41 chromosomes, 70 seeds had 42 chromosomes, four seeds had 43 chromosomes, two seeds had 44 chromosomes. Euploids and aneuploids accounted for 55.6% and 44.4% of the seeds, respectively. Within the strain of NA0928-2–1-9–1-2–2-2–2, two seeds had 38 chromosomes, six seeds had 42 chromosomes, and one seed had 43 chromosomes. Euploids and aneuploids accounted for 66.67% and 33.33% of the seeds, respectively. With regard to the strains NA0928-1–2-2–3-5–2-1–4, NA0928-2–1-10–4-1–1-1–3, and NA0928-2–1-10–4-1–3-9–9, 30 seeds were analyzed per strain; no aneuploidy was observed and all seeds had 42 chromosomes.

### FISH analysis of the S_9_ generation of NA0928

**Fig. 1:**
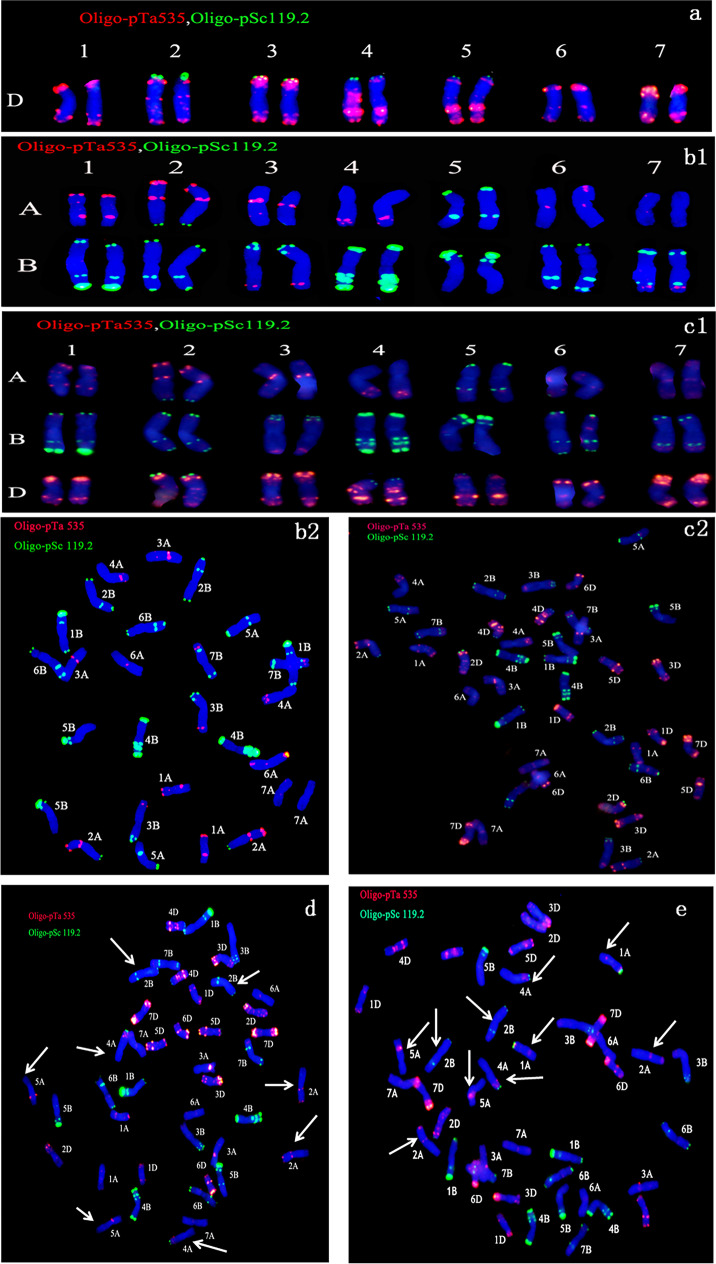
The euploid and parent configurations lines in S9 generation by FISH results. **a** Karyotype analysis of SY41; **b**1 Karyotype analysis of MY3478; **b**2 FISH patterns of MY3478; **c**1, **c**2 *2n* = 42 normal karyotype analysis and FISH patterns; **d** *2n* = 42 variation type, pSc119.2 signals (green) at the ends of chromosomes 2A, 2B, and 5A were absent, while additional green signals were observed on the long arms of chromosome 4A; **e** *2n* = 42 variation type, including the chromosomal structure variations revealed in Fig. 1d, an additional strong green signal was identified at the end of the short arm of chromosome 1A. The white arrows indicate structural variations in the chromosomes

Five seeds per plant of S_9_ strains of NA0928 were selected randomly for FISH analysis. Euploid and aneuploid chromosome configuration types were abundant. The chromosome configuration types of euploids (2*n* = 42) and aneuploids (2*n* = 40 and 2*n* = 41) were analyzed using the Oligo probes pSc119.2 and pTa535. Three types of variation with 42 chromosomes compared with the parents were observed. (1) All chromosomes showed the same FISH pattern, which was consistent with the parents (Fig. 1a, 1b1, 1b2, 1c1, 1c2). Plants of the strains NA0928-1–2-2–3-5–2-1, NA0928-2–1-9–1-2–2-2–1, and NA0928-2–1-10–2-1–3-1–1 conformed to this type. (2) The pSc119.2 signals (green) at the ends of chromosomes 2A, 2B, and 5A were absent, while additional green signals were observed on the long arms of chromosome 4A (Fig. 1d). Each plant of NA0928-2–1-10–4-1–1-1 belonged to this type. (3) Including the chromosomal structure variations revealed in the second type, an additional strong green signal was identified at the end of the short arm of chromosome 1A (Fig. 1c). Each plant of NA0928-2–1-10–4-1–3-9 adhered to this type.

**Fig. 2:**
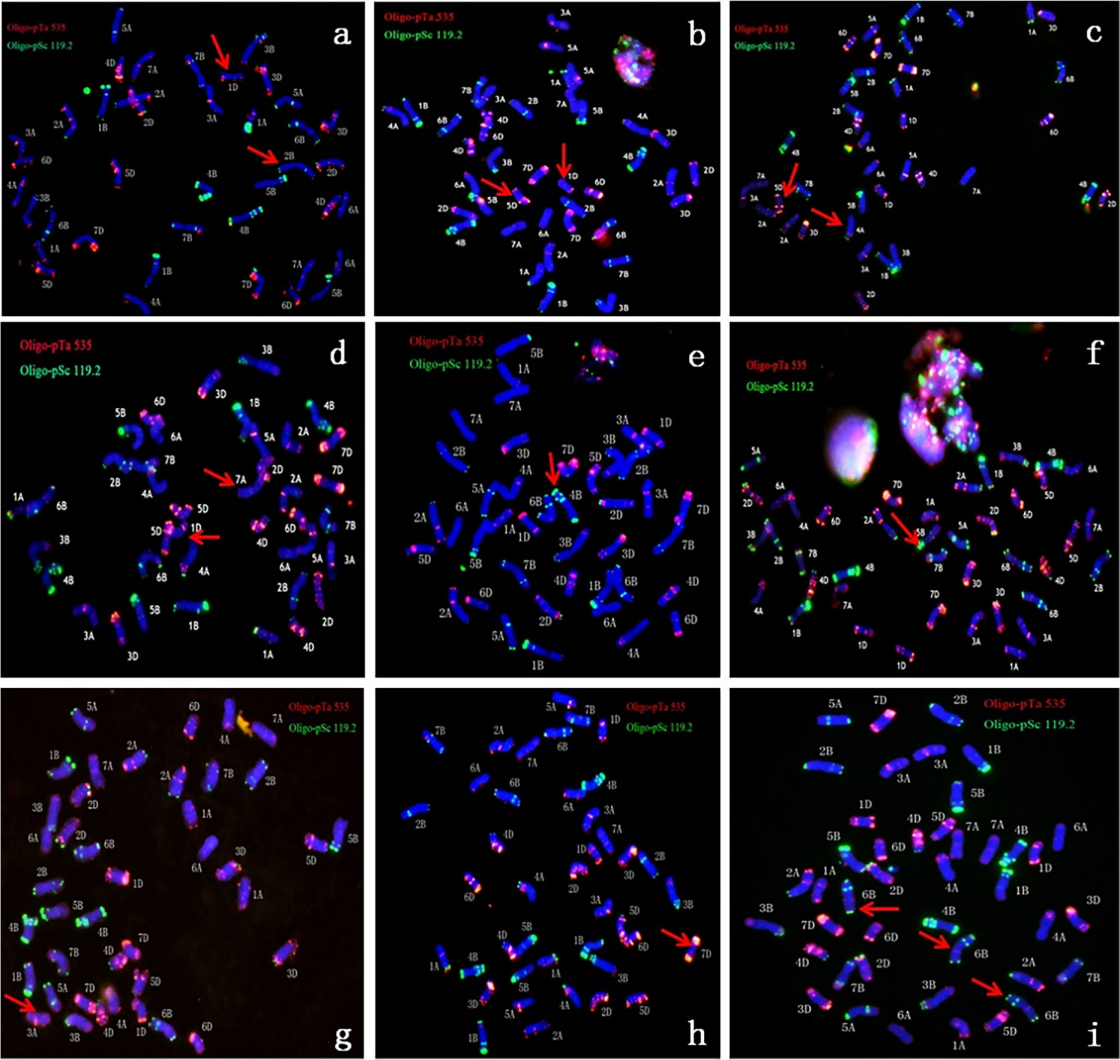
The aneuploidy configurations lines in S9 generation by FISH results. **a**, **b**, **c**, **d** 2*n* = 40; **e**, **f**, **g**, **h** 2*n* = 41; **i** 2*n* = 43. The corresponding individual respectively are the following: NA0928-1–2-2–3-5–2-1–1 (missing one chromosome 2B and 1D), NA0928-2–1-10–4-1–3-9–1 (missing one chromosome 1D and 5D), NA0928-2–1-10–4-1–3-9–2 (missing one chromosome 4A and 5D), NA0928-2–1-10–4-1–3-9–5 (missing one chromosome 7A and 1D), NA0928-1–2-2–3-5–2-1–3 (missing one 4B chromosome), NA0928-2–1-9–1-2–2-2–3 (missing one 5B chromosome, NA0928-2–1-9–1-2–2-2–4 missing one 3A chromosome, NA0928-2–1-10–2-1–3-1–4 (missing one 7A chromosome), NA0928-2–1-9–1-2–2-2–4 (three 6B chromosomes). Red arrow is missing or added chromosomes

**Fig. 3:**
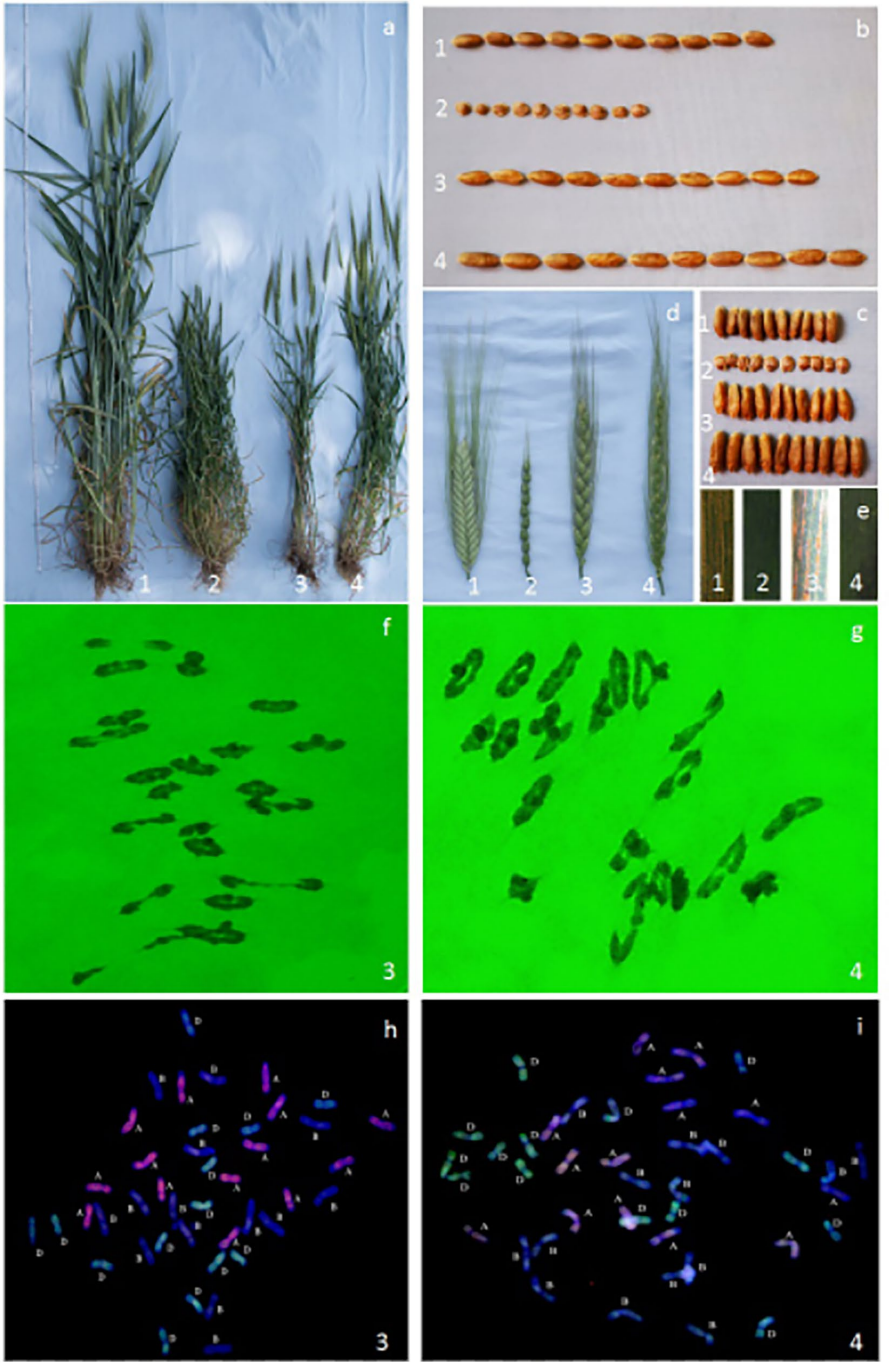
Morphological, configurations at meiotic metaphase and powdery mildew reactions of NA0928 in S9 generation. 1:CS; 2:SY159; 3:NA0928-2–1-9–1-2–2-2–1; 4:NA0928-2–1-10–4-1–1-1–3. **a** Plants; **b**,**c** Kernel; **d** Spikes; **e** Symptoms in response to inoculation with Pst races CYR32, CYR33, and CYR34 at the adult stages; **f**–**g** Configurations at meiotic metaphase; **h**–**i** mc-GISH. *Ae. tauschii* Coss. genomic DNA (green) and *Triticum urartu* Thum. genomic DNA (red) were used as probes, *Ae. Speltoides* genomic DNA was blocker, chromosomes were counterstained with DAPI (blue)

Aneuploid chromosome configuration types were abundant. Based on the same chromosome number, various chromosome configuration types were identified. Four types were characterized when the aneuploids carried 40 chromosomes: (1) missing one chromosome 2B and 1D (Fig. 2a); (2) missing one chromosome 1D and 5D (Fig. 2b); (3) missing one chromosome 4A and 5D (Fig. 2c); and (4) missing one chromosome 7A and 1D (Fig. 2d). Regarding the aneuploids containing 41 chromosomes, missing one 4B chromosome (Fig. 2e), 5B (Fig. 2f), 3A (Fig. 2g), and 7A (Fig. 2h) was identified in different individuals. The aneuploids containing 43 chromosomes carried three 6B chromosomes (Fig. 2i).

Consideration of the stripe rust resistance and FISH analysis results revealed an interesting association. The plants with a chromosome configuration identical to the parents were severely susceptible to stripe rust. However, any individual plant that showed variation in chromosome configuration compared with that of the parents was strongly resistant to stripe rust. Thus, all progeny of strains NA0928-2–1-10–4-1–1-1 and NA0928-2–1-10–4-1–3-9 were highly resistant to stripe rust.

### Two new synthetic hexaploid wheat strains with genetic stability

On the basis of the results of the FISH analysis, the plants with 42 chromosomes and a normal configuration were selected to observe meiotic metaphase in pollen mother cells. Approximately 50 pollen mother cells were observed for each strain. The pollen mother cells of two strains (NA0928-2–1-9–1-2–2-2–1 and NA0928-2–1-10–4-1–1-1–3) in the S_9_ generation showed normal meiotic chromosome behavior (2*n* = 21II) and no monoploids were observed (Fig. 3f, 3g). The results of mc-GISH showed that the chromosome composition of NA0928-2–1-9–1-2–2-2–1 and NA0928-2–1-10–4-1–1-1–3 was 14A + 14B + 14D (Fig. 3h, 3i). These results indicated that NA0928-2–1-9–1-2–2-2–1 and NA0928-2–1-10–4-1–1-1–3 were cytologically stable. The grains of these two strains were considerably larger than those of the parents (Fig. 3b, 3c); the TKW of both strains was more than 60 g. In addition, NA0928-2–1-9–1-2–2-2–1 was susceptible to stripe rust, whereas NA0928-2–1-10–4-1–1-1–3 was strongly resistant to stripe rust and developed multiple tillers (Fig. 3e). Hence, NA0928-2–1-10–4-1–1-1–3 is suitable for utilization as potential germplasm for wheat breeding.

## Discussion

Since the rise of human civilization, wheat has been an important cultivated food crop (Asseng et al. 2020). Wheat production is crucial for global food security. Improvement of yield is a primary goal of wheat breeders. It should be noted that grain yield is a function of the yield components, such as number of tillers, number of kernels per spike, and grain weight per spike (Mwadzingeni et al. 2018). Increase in grain weight is an important contributor to yield increase (Underdahl et al. 2008). In the present study, agronomic traits of certain progeny strains were superior to those of the parents, such as increased number of tillers, longer spikes, increased number of spikelets, and resistance to stripe rust. In particular, the higher TKW of NA0928-2–1-9–1-2–2-2–1 and NA0928-2–1-10–4-1–1-1–3 exceeded 60 g (Fig. 3). These two remarkable strains could be used in wheat breeding programs. The strains could be crossed with high-yielding cultivars to transfer large grain traits to generate, new germplasm resources with large grains and many favorable characters. In China, “Chuanmai 42” was the first breeding wheat cultivar derived from synthetic wheat and was authorized for national release in 2004 (no. 2004002). Our research group have crossed synthetic hexaploid wheat “SE5785” and “Xiaoyan 22,” and by selecting for agronomic traits and disease resistance over several years, the germplasm lines “N07228-1” and “N07228-2” with improved agronomic traits, large seeds, resistance to powdery mildew, and similar morphologies to common wheat were obtained (Wang et al. 2016a, 2016b). Thus, the transfer of resistance genes can broaden the narrow genetic base of wheat and expand the resistance gene pool in germplasm resource.

The cytological stability appears to break down when the increase or decrease in number of chromosomes exceeds one, which may directly result in severe aneuploid states, such as nullisomy, monosomy, trisomy, and tetrasomy. Aneuploids generally arise and are rarely recovered among the progeny of newly synthesized hexaploid wheat (Zhang et al. 2013; Zeng et al. 2020). In previous studies, aneuploid wheat was widely used for gene localization, molecular marker development, and genetic analysis (Endo and Gill, 1996; Klindworth et al. 2007; Zhang et al. 2020; Pshenichnikova et al. 2020). Chinese Spring monosomic and durum wheat (*Triticum turgidum* L. subsp. *durum* (Desf.) van Slageren) “Langdon” chromosome substitution lines are commonly used as materials for gene mapping (Sears 1953; Shimelis and Spies, 2011; Wang et al. 2020). Based on the durum wheat “Langdon” substitution lines and Abbondanza monosomic system, the crossability-related genes were located on chromosomes 1A, 6A, and 7A by Liu (1999). In the current study, several aneuploid individuals were identified by cytological identification, such as the lack of chromosomes 2B, 4B, 5B, 6B, 1D, 5D, 4A, and 7A. Based on the aneuploids, stable genetic materials can be created after several years of directional offspring selection. Such materials may be extremely useful tools for genetic analysis.

Because polyploidization is accompanied by whole-genome reorganization, most newly synthesized allohexaploid wheat suffers from cytological instability. Most of these rearrangements may be caused by chromosome pairing anomalies, which cause frequent homoeologous recombination (Marais and Charlesworth, 2003). As a result, interchromosomal exchanges and gene transformation arise and ultimately lead to the loss of a portion of the homoeologous genes. Hence, the variation in chromosome number and chromosomal structure detected in this study can be reasonably explained (Gaeta and Chris Pires, 2010). Previous studies have shown that the chromosome configuration of early generations in new synthetic hexaploid wheat is frequently unstable (Comai, 2000; Zhao et al. 2011). In the present study, the FISH patterns of NA0928 in the S_9_ generation showed structural variation in several chromosomes compared with the parents MY3478 and SY41. The pSc119.2 signals (green) at the end of chromosomes 2A, 2B, and 5A were lost, while additional green signals were observed on the long arms of chromosome 4A and the short arms of chromosome 1A. Therefore, although the chromosome number of synthetic wheat lines was similar to or identical to that of common wheat, the chromosome configuration showed many differences. The variation in chromosomal structure was detected in the A and B genomes, but no changes were observed in the D genome by FISH analysis. These results were consistent with previous findings (Pont et al. 2013; Liu et al. 2015). The two subgenomes of natural tetraploids and the three subgenomes of hexaploid wheat distinctly differ in their propensities to undergo evolutionary genomic changes; the B subgenome is more labile, the A subgenome is more stable, whereas the D subgenome is the most stable. This suggests that repeat sequences lost or restructured during the process of allopolyploidization may lead to the emergence of a novel phenotype. It could also explain the strong resistance to stripe rust exhibited by NA0928-2–1-10–4-1–1-1–1 and NA0928-2–1-10–4-1–3-9–1, which exhibited variation in chromosomal structure.

A highly interesting phenomenon was noted in the present study. The plants that showed no variation in chromosome configuration compared with the parents were strongly susceptible to stripe rust. In contrast, an individual plant that showed any variation in chromosome configuration was strongly resistant to stripe rust. Therefore, the responsible mutations must have occurred in the A and B chromosomes. However, MY3478 is severely susceptible to stripe rust, whereas SY41 is resistant to stripe rust (Fig. 3e). What was the derivation of stripe rust resistance in the hexaploid strains? In the offspring of new synthetic hexaploid wheat that showed no variation in chromosomal structure compared with the parents, the disease-resistance genes were silenced. In contrast, the offspring that showed stripe rust resistance indicated that the balance between the parental chromosomes was disrupted and the resistance genes were activated, SY41may translocation or introgression in small fragments into SHW. It suggested the resistance genes hiding within SHW can be reactivated. It also indicated the genetic background plays an important role in controlling the expression of genes in newly created SHW. The above observations suggested that using hexaploidwheats in different backgrounds as parents to cross with SHW or selection of mutant strains from the selfing population of newly created SHW were two effective means of applying SHW in wheat breeding. Considerable further research on synthetic hexaploid wheat is required, such as analysis of differences between whole-genome sequencing in early generations (S_0_ and S_1_). Although the strains that showed A and B chromosomal variation were resistant to stripe rust, their disease-resistance gene(s) may not be located on A and B chromosomes; therefore, genetic analysis is needed to verify their chromosomal location. Gene mapping has been conducted to study the genetic basis of resistance to stripe rust, grain weight, and increase in tiller number, for example. In addition, new synthetic hexaploid wheat has been hybridized with common wheat cultivars (strains) over a wide area to promote wheat breeding and utilization, and have resulted in new cultivars with larger grains, enhanced tiller production, and improved disease resistance. This approach enriches the gene pool of wheat genetic resources and expands the genetic diversity available for wheat breeding.
